# Successful Treatment of Spontaneous Coronary Artery Dissection With Cutting Balloon Angioplasty

**DOI:** 10.7759/cureus.13706

**Published:** 2021-03-04

**Authors:** Mohamed Zghouzi, Homam Moussa Pacha, Yasar Sattar, M. Chadi Alraies

**Affiliations:** 1 Internal Medicine, Detroit Medical Center, Detroit, USA; 2 Cardiovascular Disease, University of Texas Health Science Center at Houston McGovern Medical School, Houston, USA; 3 Internal Medicine, Icahn School of Medicine at Mount Sinai, New York, USA; 4 Cardiology, Detroit Medical Center, Detroit, USA

**Keywords:** spontaneous coronary artery dissection, scad, cutting balloon, endovascular angioplasty

## Abstract

Spontaneous coronary artery dissection (SCAD) is a rare but serious condition that requires immediate attention. It has a similar presentation to acute coronary syndrome in terms of chest pain, electrocardiogram changes, and an increase in troponins, and is considered to be a significant cause of myocardial infarction. Coronary angiography is needed to confirm the diagnosis, and subsequent repair should be pursued when needed. We describe a case of SCAD in a 72-year-old female treated using the cutting balloon angioplasty technique to create communication between the true and false lumens.

## Introduction

Spontaneous coronary artery dissection (SCAD) is a rare but serious condition that requires immediate attention. SCAD is an uncommon and underdiagnosed condition. It is a condition of a younger patient group and predominant in women [1,2]. It has a similar presentation to acute coronary syndrome (ACS) in terms of chest pain, electrocardiogram changes, and an increase in troponins, and is considered to be a significant cause of myocardial infarction. Coronary angiography is needed to confirm the diagnosis, and subsequent repair should be pursued when needed. The estimated prevalence of SCAD in patients presenting with ACS ranges from 1.7 to 4% [3,4], and in women younger than 50 years of age and presenting with ACS, it ranges from 9 to 43% [4-6]. Once the diagnosis is confirmed, management is often determined by the angiographic patency of the coronary artery. Options for therapy include conservative management with no intervention, coronary angioplasty, or coronary stenting. However, coronary imaging could establish the diagnosis and etiology. Therefore, the current recommendation is to refrain from coronary stenting, especially if the coronary flow is preserved. In this case, we present a patient with ACS who was found with coronary dissection and treated with cutting balloon angioplasty alone. To confirm the patency of the coronary artery, computed tomography angiogram was done a few weeks later, which showed patency of the vessel.

## Case presentation

**Figure 1:**
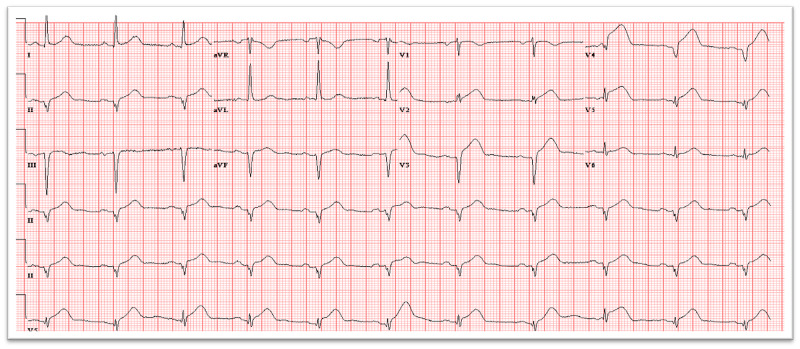
ECG showing diffuse ST-segment elevation in leads V1-V5. ECG, electrocardiogram

A 72-year-old female with a medical history of hypertension, paroxysmal atrial fibrillation, type B thoracic aortic dissection status, post-percutaneous endovascular repair, stenting gastroepiploic artery aneurysm status post coil embolization, and mitral valve prolapse presented with chest pain. She described the chest pain as heavy and centrally located. The chest pain had progressed in severity by the time she was seen in the emergency department. ECG showed diffuse ST-segment elevation (Figure 1).

Bedside ECG was performed, showing an ejection fraction of 25%, severely hypokinetic anterior wall, and an akinetic apical septum, apex, and inferior wall. Laboratory investigation demonstrated a troponin level of 1 ng/mL.

**Figure 2:**
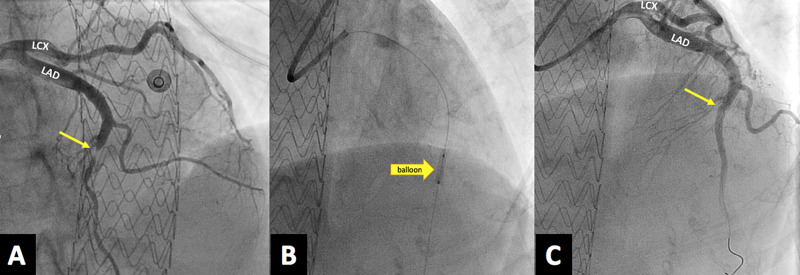
(A) Complete occlusion of the mid-section of the LAD artery and a TIMI flow score of 0-1 due to SCAD (arrow). (B) Coronary angiography showing 2.5-mm cutting balloon dilated in the proximal and distal segments of LAD artery (arrow). (C) Coronary angiography showing coronary flow restored to TIMI flow 3 after ballooning (arrow). LAD, left anterior descending; TIMI, Thrombolysis in Myocardial Infarction; SCAD, spontaneous coronary artery dissection

The patient was taken emergently to the cardiac catheterization laboratory. Radial access and a 5-Fr tiger catheter were used. Complete occlusion of the mid-section of the left anterior descending (LAD) artery and Thrombolysis in Myocardial Infarction (TIMI) flow score of 0-1 due to spontaneous coronary artery dissection (Figure 2A) were noted. A 0.014-Fr Fielder XT coronary guidewire was advanced across the dissection planes, and the mid-LAD high-grade stenosis was achieved without difficulty. Although the initial plan was to stent the mid-LAD stenotic segment, as the lesion was not atherosclerotic, a decision to perform atherectomy balloon angioplasty of the segment using the 3.0 × 20 mm “cutting balloon” was made (Boston Scientific, Marlborough, MA, USA) (Figure 2B). Cutting balloon angioplasty created communication between the true and false lumens and restored the distal coronary flow (Figure 2C).

After the procedure, the TIMI flow score was restored to 3. The procedure was uncomplicated, and the patient was discharged in good condition on warfarin and clopidogrel, given her history of paroxysmal atrial fibrillation.

**Figure 3:**
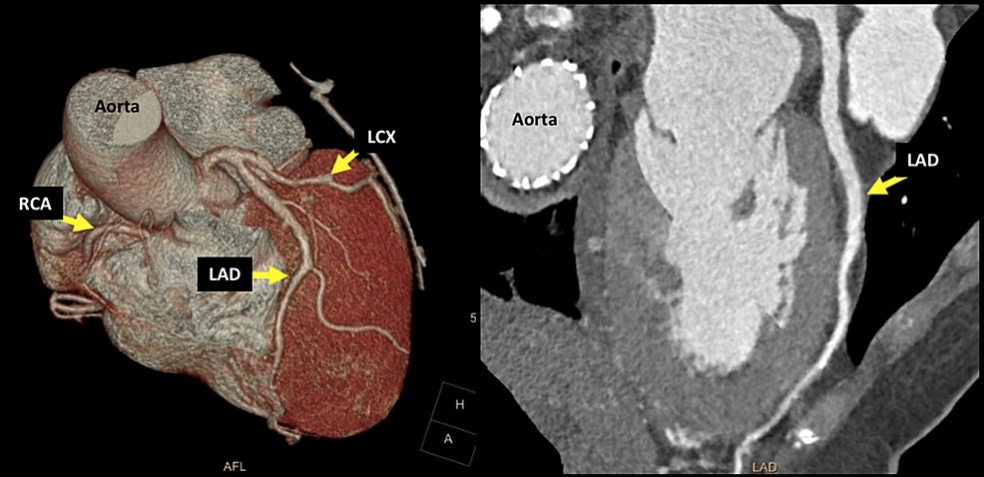
CTA showing ordinary course and caliber of LAD artery fully opacified without luminal narrowing or stenosis or evidence of dissection. CTA, computed tomography angiography; LAD, left anterior descending; LCX, left circumflex artery; RCA, right coronary artery

A four-week follow-up coronary computed tomography was performed, showing a normal course and caliber of the LAD artery giving rise to two diagonal branches. Furthermore, the vessel was fully opacified throughout without luminal narrowing, stenosis, or evidence of dissection (Figure 3).

## Discussion

SCAD is defined as a spontaneous separation of the coronary artery wall that is not iatrogenic or related to trauma [2]. The first reported case of SCAD was in 1931 in middle-aged women.

Usually, patients present with an ACS spectrum such as unstable angina, non-ST elevation myocardial infarction, ST-elevation myocardial infarction, or sudden cardiac death [1]. The etiology is nonatherosclerotic and associated with clinical features such as fibromuscular dysplasia, pregnancy or postpartum state, connective tissue disorders, systemic inflammatory disease, and coronary artery spasm. Precipitators usually include exertion or emotional stress [1,2]. Many SCAD patients have a low pre-test probability of coronary artery disease, potentially missing the diagnosis or delayed intervention.

Coronary angiography is the golden standard for diagnosing SCAD. Furthermore, intracoronary imaging made the diagnosis of SCAD easy in patients presenting with ACS. This condition is usually sub-categorized into three types: type 1 (contrast staining delineating multiple lumens), type 2 (diffuse smooth narrowing), and type 3 (similar to atherosclerosis), with type 2 being the most common. The use of intracoronary in the form of optical coherence tomography and intravascular ultrasound can help diagnose type 2 and type 3 ACS [2,7].

The management of acute SCAD depends on the affected vessel’s distal flow, clinical, and hemodynamic stability. Percutaneous coronary intervention (PCI) is often challenging and is associated with a high failure rate, even when distal coronary perfusion is preserved [8]. Medical management is reserved for clinically stable patients with a good distal coronary flow (TIMI score: 2-3) [8]. When hemodynamic stability is not achieved or in the case of failed medical management, PCI or coronary artery bypass grafting is indicated. There are several cases reported in the literature where scoring/cutting balloon angioplasty is used to treat SCAD, either as an initial treatment or when stenting failed to restore flow to the affected artery (Table 1).

There are scant data concerning the ideal medical therapy for acute SCAD. Aspirin is given to most patients, regardless of their atherosclerotic disease status. Clopidogrel and heparin are reserved for clinically appropriate situations. Beta-blockers play an important role in reducing arterial shear stress and ventricular arrhythmia and improving survival [2,9]. Angiotensin-converting enzyme inhibitors treatment is reserved for patients with left ventricular dysfunction. Treatment with nitrates or calcium channel blockers is reasonable in the presence of chest pain, which is likely related to dissection or coronary spasm.

Long-term survival in acute SCAD settings is excellent, but the risk of recurrence and myocardial infarction is high in this patient population. [1] Daily low-dose aspirin is recommended for all patients, but dual antiplatelet therapy is reserved for patients with PCI.

**Table 1:** Previous case reports in the literature describing cutting/scoring balloon angioplasty in SCAD treatment. LAD, left anterior descending; CTA, computed tomography angiography; RCA, right coronary artery; ECG, electrocardiogram; LM, left main; LCX, left circumflex artery; SCAD, spontaneous coronary artery dissection

Author	Year	Age	Sex	Location	Treatment	Imaging follow-up
Terzian et al. [10]	2019	34	F	LAD	Scoring balloon angioplasty	Coronary angiogram at 9 months
Kaya et al. [11]	2019	46	F	LAD	Cutting balloon angioplasty	Coronary CTA at 1 and 6 months
Sharma et al. [12]	2019	53	F	RCA	Cutting balloon angioplasty	NA
Main et al. [13]	2019	62	F	Large diagonal branch	Cutting balloon angioplasty	ECG in 6 weeks
Bresson et al. [14]	2019	36	F	Proximal LAD	Stenting and cutting balloon angioplasty	NA
McGrath et al. [15]	2018	51	F	LM, LAD, LCX	Stenting and cutting balloon angioplasty	NA
Noguchi et al. [16]	2018	42	M	LAD, LCX	Cutting balloon angioplasty and stenting	Coronary angiogram at 6 months
Ito et al. [17]	2017	46	F	LAD	Cutting balloon angioplasty	Coronary CTA at 3 months
Alkhouli et al. [18]	2016	50	F	LAD	Cutting balloon angioplasty and stenting	NA
Yumoto et al. [19]	2014	47	F	LAD	Cutting balloon angioplasty	Coronary angiogram at 6 months
Uema et al. [20]	2013	42	F	LAD	Cutting balloon angioplasty	NA

## Conclusions

Balloon angioplasty using a cutting balloon for patients presenting with SCAD and TIMI score 0 flow can offer immediate and long-term flow patency of a coronary artery with no requirement for coronary stenting.
